# Active vs. sham pulsed electromagnetic field therapy added to standardized conservative care for partial-thickness supraspinatus tears: study protocol for a randomized, assessor-blinded, sham-controlled trial

**DOI:** 10.3389/fspor.2026.1847412

**Published:** 2026-07-15

**Authors:** Tianzhu Du, Xu Hu, Yu Dan, Yu Zhou, Hongyuan Liu, Fei Wang, Jun Tao

**Affiliations:** 1 Department of Rehabilitation Medicine, TCM Hospital of Junlian, Yibin, Sichuan, China; 2 Department of Rehabilitation Medicine, Yibin Hospital of Traditional Chinese Medicine, Yibin, Sichuan, China; 3 SCATCM Key Laboratory of Traditional Chinese Medicine Health Products Research and Clinical Evaluation (Yibin Traditional Chinese Medicine Hospital), Yibin, Sichuan, China; 4 Yibin Clinical Research Center for Traditional Chinese Medicine Health Products, Yibin, Sichuan, China; 5 Department of Orthopedics, Yibin Hospital of Traditional Chinese Medicine, Yibin, Sichuan, China

**Keywords:** partial-thickness tear, pulsed electromagnetic field, randomized controlled trial, sham-controlled trial, shoulder rehabilitation, supraspinatus tear

## Abstract

**Background:**

Partial-thickness supraspinatus tears commonly cause shoulder pain and functional limitation. Although conservative treatment can improve symptoms, whether pulsed electromagnetic field (PEMF) therapy provides additional clinically meaningful benefit remains uncertain. Existing PEMF evidence is mainly derived from rotator cuff tendinopathy or subacromial impingement populations, whereas sham-controlled evidence in MRI-confirmed partial-thickness supraspinatus tears remains sparse.

**Methods:**

This is a single-center, parallel-group, randomized, assessor-blinded, sham-controlled trial. Fifty adults with symptomatic MRI-confirmed partial-thickness supraspinatus tears and baseline activity-related shoulder pain of at least 3 cm on a 10-cm Visual Analog Scale (VAS) will be recruited at Yibin Hospital of Traditional Chinese Medicine and randomized 1:1 to active PEMF plus standardized conservative care or sham PEMF plus identical standardized conservative care. Standardized conservative care includes oral nonsteroidal anti-inflammatory drugs when indicated, low-frequency pulsed electrical stimulation, activity modification, and a structured home exercise program. Active PEMF will be delivered using the YS2002 device (30 mT, 40 Hz) for 20 min once daily for 4 weeks; the sham procedure will be identical in appearance, treatment schedule, indicator lights/sounds, heat, vibration, and staff interaction but will not deliver an effective electromagnetic field. The randomized contrast therefore isolates the electromagnetic-field component of the YS2002 platform, because all other conservative-care elements, heat/vibration settings, and staff interactions will be standardized in both groups. Outcomes will be assessed at baseline, Week 4, and at 3, 6, and 12 months. The primary outcome is the between-group difference in Constant-Murley Score at Week 4 adjusted for baseline score, defined as an early clinical functional endpoint rather than a measure of structural tendon healing. Secondary outcomes include pain, range of motion, Shoulder Pain and Disability Index, MRI structural outcomes at 6 months, adverse events, adherence, and additional shoulder-related interventions during follow-up.

**Discussion:**

This trial will provide sham-controlled evidence on whether active electromagnetic-field delivery provides additional short-term symptomatic and functional benefit and acceptable safety when added to a standardized conservative-care package for symptomatic partial-thickness supraspinatus tears. The 6-month MRI outcomes and longer-term clinical outcomes will be interpreted as exploratory signals to inform future multicenter trials rather than definitive evidence of biological repair.

**Trial Registration:** https://www.chictr.org.cn/showproj.html?proj=307249, identifier ChiCTR2600119386.

## Introduction

Rotator cuff tears are a common cause of shoulder pain and disability and account for roughly 17%–41% of shoulder disorders (1). The prevalence of rotator cuff tears rises substantially with age, from a relatively low frequency in younger adults to more than 50% in individuals older than 80 years (1). Patients typically suffer persistent shoulder pain (often worse at night), restricted range of motion, and reduced strength, which significantly impact daily activities, work, and sleep and place a burden on patients, families, and healthcare systems (1). For partial-thickness tears and many small-to-moderate tears, particularly in older or lower-demand patients, nonoperative treatment is generally considered first-line management, whereas surgery is typically reserved for selected cases with larger tears, persistent symptoms, or failure of adequate conservative care (2). However, the long-term structural course of conservatively managed partial-thickness tears is variable rather than uniform. Imaging follow-up studies suggest that some symptomatic tears enlarge or progress to full-thickness defects over time, whereas others remain structurally stable, indicating persistent uncertainty regarding which patients are most likely to deteriorate (3, 4). Although arthroscopic repair is effective for selected cases, it entails anesthesia risks, prolonged rehabilitation, substantial costs, and re-tear rates that remain a concern (2). Consequently, optimizing evidence-based non-surgical treatment remains clinically important, particularly for patients in whom surgery is not initially indicated or is undesirable (2, 5, 6).

Current conservative management for partial rotator cuff tears typically combines analgesic or anti-inflammatory medication with rehabilitation-based care. In the present trial, standardized conservative care includes oral NSAIDs when indicated, low-frequency pulsed electrical stimulation, activity modification, and a structured home exercise program. More broadly, common nonoperative approaches also include exercise-based rehabilitation, physical modalities, and, in some settings, complementary approaches such as acupuncture, although the exact content of care varies across clinical settings and practice recommendations (6, 7). Although these measures often reduce pain and improve function, evidence for consistent structural tendon restoration is limited, and imaging follow-up suggests that a subset of symptomatic tears may enlarge despite nonoperative care (3–5). Moreover, current conservative options have important limitations: long-term NSAID use is constrained by gastrointestinal, cardiovascular, and renal risks (8, 9), recommendations for physical and rehabilitation modalities vary across shoulder guidelines (10), and exercise-based rehabilitation requires sustained adherence over time (11). Therefore, a safe and well-tolerated adjunctive therapy that can promote tissue repair in addition to symptom control remains desirable (7).

Preclinical studies provide biologic plausibility for investigating PEMF as an adjunct to rehabilitation. In rat rotator cuff injury and repair models, PEMF exposure has been associated with improved collagen organization, fibrocartilage formation, and tendon-to-bone biomechanical properties; frequency and exposure duration may influence the magnitude of these effects (12–14). These data support a potential mechanism for tendon-related effects but do not establish clinical efficacy or an optimal regimen for nonsurgically managed partial-thickness supraspinatus tears.

Pulsed electromagnetic field (PEMF) therapy is a non-invasive biophysical modality investigated for analgesic and tissue-healing effects in musculoskeletal disorders. Clinical shoulder evidence is encouraging but heterogeneous and is concentrated mainly in rotator cuff tendinopathy and subacromial impingement syndrome rather than structurally defined partial-thickness supraspinatus tears (15–18). Randomized and sham-controlled shoulder studies have reported potential short-term improvements in pain and function, and a recent synthesis suggested functional benefit in subacromial impingement, but device settings, co-interventions, and study populations varied substantially (15–18).

Evidence directly addressing supraspinatus tendon tears remains sparse and inconsistent. In one randomized study involving patients with supraspinatus tendon tears, adding PEMF to conventional physical therapy did not demonstrate superiority over control treatment (19). A small body of Chinese clinical literature has reported potential improvements when PEMF or related magnetotherapy was combined with other rehabilitation modalities for rotator cuff injury (20–22), but these studies used heterogeneous designs, co-interventions, device parameters, and outcome assessments. Therefore, a sham-controlled trial in a clearly defined MRI-confirmed supraspinatus partial-thickness tear population remains warranted. To address this gap, we designed an assessor-blinded randomized trial comparing active PEMF plus standardized conservative care with sham PEMF plus identical standardized conservative care. The study incorporates multidimensional outcomes and 12-month follow-up to evaluate early clinical response, durability, and exploratory structural signals.

## Methods and analysis

### Objectives

This trial has one primary objective and several secondary objectives:

- Primary Objective: To evaluate whether active pulsed electromagnetic field (PEMF) therapy in addition to standardized conservative care leads to superior early improvement in shoulder function compared with sham PEMF plus identical standardized conservative care at Week 4 in adults with MRI-confirmed partial-thickness supraspinatus tears. The Week 4 primary endpoint will be interpreted as a short-term clinical and symptomatic endpoint rather than evidence of structural tendon healing.
- Secondary Objectives:
- Evaluate the effect of PEMF on shoulder pain intensity (Visual Analog Scale, VAS) at Week 4 and the durability of pain relief at 3, 6, and 12 months compared with sham PEMF.
- Compare changes in shoulder range of motion (ROM) between the active PEMF and sham PEMF groups at Week 4 and during follow-up at 3, 6, and 12 months.
- Assess exploratory changes in MRI structural measures of the partial-thickness supraspinatus tear from baseline to 6 months between groups, including tear location, maximal depth, maximal anterior-posterior length, estimated tendon-thickness involvement, and progression to full-thickness tear.
- Monitor and compare adverse events, treatment adherence, drop-outs, and longer-term clinical trajectories between groups, including the proportion of participants requiring additional interventions (e.g., corticosteroid injection or surgery) during the 12-month follow-up. Structural MRI outcomes and longer-term treatment trajectories will be interpreted as secondary exploratory endpoints intended primarily to generate effect-size estimates and mechanistic signals for future trials.

### Trial design

This study is a single-center, randomized, sham-controlled, assessor-blinded, parallel-group superiority trial with a 1:1 allocation ratio, conducted at Yibin Hospital of Traditional Chinese Medicine. The planned recruitment period is April 2026 to February 2028. Treatment providers will not participate in outcome assessment. The trial is designed to estimate the incremental effect of active electromagnetic-field delivery because NSAID use, electrical stimulation, activity advice, home exercise, heat, vibration, session duration, and therapist interaction will be matched between groups.

The protocol was prepared in accordance with the SPIRIT 2025 statement (23).

### Sponsor and trial governance

Sponsor: Yibin Hospital of Traditional Chinese Medicine, Yibin, Sichuan, China. Sponsor contact: Guobing Wang, Yibin Hospital of Traditional Chinese Medicine, moonly1981@163.com.

Sponsor role: The sponsor will have no role in data collection, data management, data analysis, data interpretation, or manuscript preparation, and will have no influence on the decision to submit the results for publication. The trial management group will oversee recruitment, intervention fidelity, data quality, protocol adherence, and safety reporting.

### Study setting

All trial-related treatments and assessments will be conducted on an outpatient basis in the Department of Rehabilitation Medicine, Yibin Hospital of Traditional Chinese Medicine.

### Patient and public involvement

No patient or public involvement is planned in the design, conduct, or reporting of this trial.

### Participant protections and informed consent

The study will be conducted in accordance with the Declaration of Helsinki. The protocol was approved by the Ethics Committee of Yibin Hospital of Traditional Chinese Medicine [approval no. KY2026年审(003)号]. All participants will provide written informed consent before any study-specific procedures are undertaken.

No biological specimens will be collected and no ancillary studies are planned; therefore, no additional consent provisions are required.

Ancillary and post-trial care: Participants will continue to receive routine clinical care as needed, and any additional treatments during follow-up will be documented. Compensation: No additional financial compensation is planned for participation. If any harm related to trial participation occurs, it will be managed by the hospital in accordance with local regulations and institutional policies.

### Eligibility criteria

Inclusion criteria: Patients must meet all of the following:

- Age between 40 and 70 years old (inclusive).
- Clinical presentation of shoulder pain and limited shoulder mobility (or difficulty elevating the arm) with symptom duration of ≤3 months.
- Baseline activity-related shoulder pain intensity of at least 3 cm on a 10-cm Visual Analog Scale (VAS), assessed during shoulder elevation or other usual provoking shoulder activity over the preceding 7 days.
- MRI confirmation of a partial-thickness tear confined to the supraspinatus tendon, characterized by tear location (articular-sided, bursal-sided, or intrasubstance), maximal tear depth, maximal anterior-posterior tear length, and estimated tendon-thickness involvement. The maximal anterior-posterior tear length must be ≤3 cm on baseline MRI.
- Unilateral shoulder involvement (only one shoulder affected).
- Able and willing to provide informed consent and to comply with the treatment protocol and follow-up assessments.

Exclusion criteria: Patients meeting any of the following:

- Presence of other significant shoulder pathologies such as adhesive capsulitis (frozen shoulder), calcific tendinitis, or a history of significant shoulder trauma or surgery on the affected side.
- Previous receipt of treatments similar to the study interventions for the current shoulder condition since symptom onset (e.g., prior PEMF therapy, steroid injections, or other formal therapy specifically for this tear, which could confound results).
- Open wounds, dermatologic lesions, or active infection over the affected shoulder region (which could interfere with safe application of therapies).
- Severe underlying medical conditions, including serious hepatic or renal impairment or uncontrolled cardiovascular disease, that would pose excessive risk to the patient or confound outcome assessments.
- Contraindications to MRI or device-based therapy, including a cardiac pacemaker or other implanted electronic device, or other device-specific contraindications according to the manufacturer's instructions for use.

Discontinuation of study treatment and withdrawal from follow-up: Participants may discontinue the assigned study treatment at any time for safety, tolerability, personal preference, or clinician judgment, while still remaining in follow-up unless they withdraw consent for outcome collection. Investigators may discontinue the study intervention if continued participation poses unacceptable risk or if post-randomization information reveals an exclusion criterion. Major non-adherence (for example, failure to complete at least 20 of the 28 planned YS2002 sessions) and receipt of prohibited co-interventions during the 4-week intervention period will be recorded as protocol deviations and considered in per-protocol and sensitivity analyses, but will not automatically result in withdrawal from the trial.

### Sample size

Using PASS 15.0 software, we calculated that 23 participants per group would be required to detect a between-group difference of 10 points in the Constant-Murley Score (CMS) at Week 4, assuming a common standard deviation of 12 points, a two-sided alpha level of 0.05, and 80% power. The 10-point target difference was informed by published CMS minimal clinically important difference estimates in rotator cuff surgery populations (24) and was adopted here as a pragmatic clinically relevant planning effect. Because directly comparable variance estimates from an identical trial population were unavailable, the standard deviation assumption should be interpreted as a pragmatic planning parameter rather than an empirically fixed value. Because the trial is powered for a 10-point Week 4 CMS difference, it may be underpowered to detect smaller but potentially worthwhile clinical effects and is not powered to detect meaningful between-group differences in MRI structural outcomes. Allowing for approximately 8%–9% attrition before the Week 4 primary endpoint assessment, we plan to enroll 25 participants per group (50 total). Extended follow-up to 12 months may incur additional attrition; therefore, we will use retention strategies and statistical methods that accommodate missing repeated-measures data. Longer-term and structural outcomes will be analyzed as secondary exploratory outcomes and will provide effect-size estimates for future multicenter trials.

### Recruitment and allocation

A multidisciplinary team of physicians (including orthopedic surgeons and rehabilitation specialists) will screen potential participants to identify those who meet the inclusion criteria and none of the exclusion criteria. Eligible patients will receive written and verbal study information, and written informed consent will be obtained before any study-specific procedures. Eligibility may be established using a clinically indicated shoulder MRI performed within 6 weeks before enrollment if the scan meets the protocol imaging requirements, includes protocol-compatible sequences, and satisfies the minimum image quality criteria specified in the trial imaging manual; otherwise, a baseline MRI will be obtained after consent and before randomization. Baseline assessments will then include demographic and clinical data, baseline outcome measures, and baseline MRI structural measurements.

Enrolled participants will then be randomly assigned to either the active PEMF group or the sham PEMF group in a 1:1 ratio. The randomization sequence will be generated using computer-generated permuted blocks (variable block sizes of 4 and 6) by an independent researcher who is not involved in recruitment, treatment delivery, outcome assessment, or data analysis. The block sizes will not be disclosed to recruiting staff. The allocation sequence will be implemented using sequentially numbered, opaque, sealed envelopes prepared by the independent researcher. After a participant has completed baseline assessment and been confirmed eligible for randomization, the next envelope in sequence will be opened by an unblinded treatment coordinator, who will configure the YS2002 device in active or sham mode for that participant.

To minimize performance and expectation bias, this trial will use a sham PEMF control. Participants will be informed that they will receive either active or sham PEMF in addition to standardized conservative care, but they will not be told which mode is being delivered. Outcome assessors, MRI readers, and the statistician will remain blinded to treatment allocation. Treatment sessions will be delivered by trained rehabilitation staff using a standardized interaction script; because these staff operate the assigned device mode, they will not participate in outcome assessment or data analysis. Before recruitment begins, active and sham modes will be bench-tested using a magnetic-field detector/gaussmeter, with settings checked against the study intervention manual and device operating range, to verify that the sham mode does not deliver an effective electromagnetic field while preserving matched appearance, session duration, indicator lights/sounds, thermal setting, vibration setting, and staff interaction. At the Week 4 visit, participants will be asked to indicate whether they believe they received active PEMF, sham PEMF, or do not know. Blinding success will be evaluated descriptively and, where appropriate, using the Bang blinding index (25).

Emergency unblinding will be permissible only when knowledge of treatment allocation is essential for the clinical management of a serious adverse event or other urgent medical situation. Any unblinding will be authorized by the principal investigator, documented with the date and reason, and reported according to institutional requirements.

### Participant timeline

**Figure 1:**
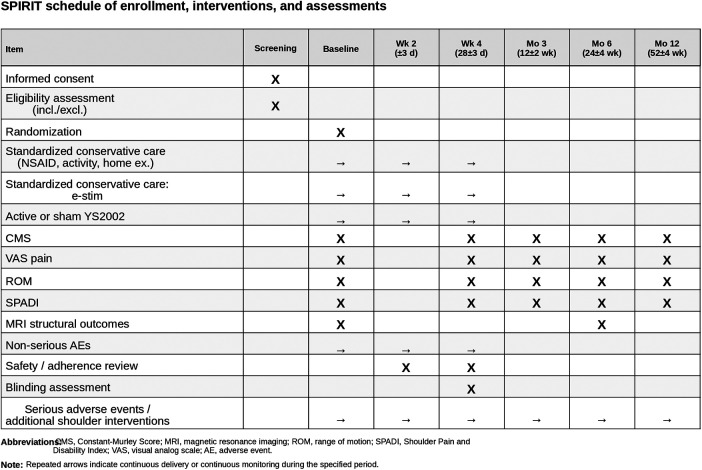
SPIRIT schedule of enrollment, interventions, and assessments. Schedule of screening, baseline assessment, allocation, intervention delivery, safety and adherence review, primary endpoint assessment at Week 4, and follow-up assessments through Month 12. CMS, Constant-Murley Score; MRI, magnetic resonance imaging; ROM, range of motion; SPADI, Shoulder Pain and Disability Index; VAS, visual analog scale.

**Figure 2:**
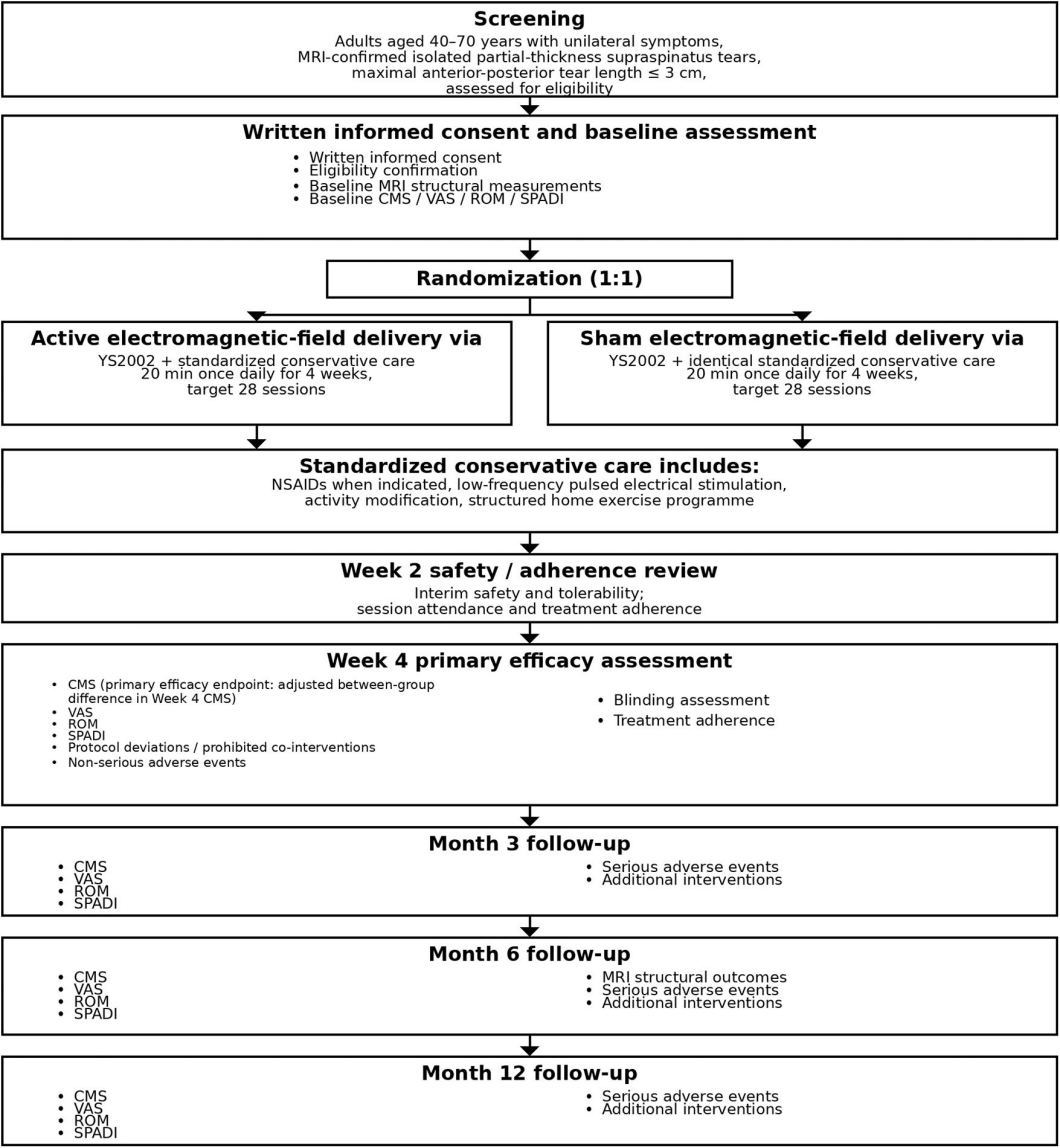
Trial flow diagram. Adults aged 40–70 years with unilateral symptomatic, MRI-confirmed isolated partial-thickness supraspinatus tears and baseline activity-related VAS pain ≥3 cm are screened, randomized 1:1 to active or sham YS2002 electromagnetic-field delivery plus identical standardized conservative care for 4 weeks, and followed at Week 4 and at Months 3, 6, and 12.

The SPIRIT schedule of enrollment, interventions, and assessments is shown in Figure 1 and Table 1, and the overall trial flow is shown in Figure 2.

**Table 1:** Schedule of enrollment, interventions, and assessments.

Item	Screening	Baseline	Wk 2 (±3 d)	Wk 4 (28 ± 3 d)	Mo 3 (12 ± 2 wk)	Mo 6 (24 ± 4 wk)	Mo 12 (52 ± 4 wk)
Informed consent	X						
Eligibility assessment (incl./excl.)	X						
Randomization		X					
Standardized conservative care (NSAID, activity, home ex.)		→	→	→			
Standardized conservative care: e-stim		→	→	→			
Active or sham YS2002		→	→	→			
CMS		X		X	X	X	X
VAS pain		X		X	X	X	X
ROM		X		X	X	X	X
SPADI		X		X	X	X	X
MRI structural outcomes		X				X	
Non-serious AEs		→	→	→			
Safety/adherence review			X	X			
Blinding assessment				X			
Serious adverse events/additional shoulder interventions		→	→	→	→	→	→

CMS, Constant-Murley Score; ROM, range of motion; SPADI, Shoulder Pain and Disability Index; VAS, visual analog scale; AE, adverse event; SAE, serious adverse event. Repeated arrows indicate continuous delivery or continuous monitoring during the specified period.

### Participant retention and follow-up procedures

A brief Week 2 contact or visit will be used to review treatment adherence, tolerability, and interim safety issues.

To maximize retention through 12 months, follow-up visits will be scheduled at the time of enrollment and confirmed via telephone or secure messaging reminders. Participants will be offered predefined visit windows and flexible scheduling. At baseline, at least two contact methods will be recorded. Discontinuation of study treatment will not, by itself, terminate follow-up. If an in-person follow-up assessment is not feasible, prespecified patient-reported outcomes and information on interim treatments and adverse events will be collected remotely whenever possible. Objective assessments requiring direct examination, including CMS strength testing, shoulder ROM measurement, and the 6-month MRI, will be performed in person whenever feasible and otherwise recorded as missing for that time point.

### Interventions

All participants will receive standardized conservative care for the shoulder for 4 weeks. The standardized conservative-care package comprises a prespecified oral NSAID regimen when indicated, low-frequency pulsed electrical stimulation, standardized advice on activity modification, and a structured home exercise program. The NSAID algorithm prespecifies ibuprofen or celecoxib as the oral options according to contraindications and tolerability, with topical NSAID use permitted when oral treatment is unsuitable. Low-frequency pulsed electrical stimulation will be delivered to the affected shoulder for approximately 20 min once daily for 4 weeks using a standardized settings framework defined in the intervention manual for device type, frequency, pulse width, intensity range, electrode placement, and session schedule. The home exercise component is limited during the 4-week intervention phase to gentle pendulum and pain-limited range-of-motion exercises rather than progressive strengthening, as specified below. In addition, all participants will receive daily treatment using the YS2002 magnetic thermo-vibration therapy device applied over the affected supraspinatus region. Heat and vibration settings will be standardized and identical in both groups; therefore, the randomized treatment contrast is active vs. sham electromagnetic-field delivery, not the entire multimodal rehabilitation package. All treatment delivery and any tolerability-related deviations will be documented on standardized session logs.

Home exercise program: The structured home exercise program will be taught face-to-face at baseline by a rehabilitation therapist, supported by a written illustrated exercise sheet, and recorded in a patient diary. During the 4-week intervention phase, exercises will emphasize pain-limited mobility and low-load scapular control rather than resisted rotator-cuff strengthening. The program will include: (1) pendulum/Codman exercises in flexion-extension, medial-lateral, and clockwise/counter-clockwise directions with the trunk slightly flexed and the affected arm relaxed; (2) active-assisted shoulder forward flexion/scaption and abduction using the unaffected arm, table slides, or wall slides; (3) active-assisted external rotation with the elbow at the side and flexed to 90 degrees, using a towel roll if needed; and (4) gentle scapular setting/retraction-depression exercises targeting low-load activation of the periscapular stabilizers. Participants will perform the program twice daily, approximately 10–15 min per session. Unless pain or fatigue requires reduction, each session will include pendulum movements for 30–60 s in each direction and 2 sets of 10 repetitions for each range-of-motion or scapular-control exercise. Progression during the 4 weeks will be limited to increasing the pain-free arc and moving from supported/table-assisted to wall-assisted active-assisted movement when pain during exercise remains ≤3/10 and there is no next-day symptom exacerbation. Resisted strengthening, loaded overhead exercise, and high-velocity stretching will be deferred until after the Week 4 primary endpoint assessment.

All electrical stimulation and PEMF/sham PEMF sessions will be administered by rehabilitation department staff trained in the standardized intervention protocol, intervention manual, and device safety procedures.

Rationale for PEMF parameter selection: The optimal PEMF dose for nonsurgical partial-thickness supraspinatus tears is not established. The 30 mT, 40 Hz, 20-minute daily regimen was selected *a priori* to provide a reproducible, feasible outpatient dose within the YS2002 device operating range while remaining broadly consistent with biologically plausible ranges in the shoulder PEMF literature. Preclinical rotator cuff models suggest that PEMF can influence early tendon-to-bone healing and that frequency and exposure duration affect healing responses (12–14). Clinical shoulder trials and meta-analytic data show heterogeneity in PEMF parameters. Some clinical studies using higher-intensity settings have reported favorable functional outcomes in shoulder impingement populations, although formal dose-response comparisons and head-to-head comparisons of different intensities are lacking (15–18). Thus, 30 mT was selected as a standardized higher-intensity device output, 40 Hz as a low-frequency setting within the commonly reported clinical range, and 20 min once daily as a pragmatic schedule compatible with routine outpatient delivery. These settings should be interpreted as prespecified, standardized trial parameters rather than a proven optimal biological dose for tendon repair.

- Control Group (Sham PEMF + Standardized Conservative Care): Participants in the control arm will receive a standardized 4-week conservative care package consisting of a prespecified oral NSAID regimen when not contraindicated, low-frequency pulsed electrical stimulation to the affected shoulder, standardized advice on rest and activity modification, and a structured home exercise program. Oral NSAID therapy will follow the prespecified departmental study protocol; ibuprofen or celecoxib will be selected according to contraindications, tolerability, and routine clinical appropriateness, with prespecified dose, frequency, and duration recorded for each participant. If oral NSAIDs are contraindicated or not tolerated, a topical NSAID may be used according to the same protocol. Low-frequency pulsed electrical stimulation will be delivered to the affected shoulder for approximately 20 min once daily for 4 weeks using the departmental protocol and intervention-manual settings for device type, frequency, pulse width, intensity, and electrode placement; the same schedule and settings framework will be applied consistently across participants unless safety or tolerability requires modification. Participants will additionally receive sham electromagnetic-field sessions using the YS2002 magnetic thermo-vibration therapy device once daily for 4 weeks (target: 28 sessions; prespecified minimum adherence threshold for per-protocol analysis: 20 completed sessions). The sham procedure will mimic the active procedure in positioning, device appearance, session duration, indicator lights/sounds, thermal setting, vibration setting, and staff interaction, but will deliver no effective electromagnetic field. Before trial initiation, the sham configuration will be bench-tested using a magnetic-field detector/gaussmeter, with settings checked against the study intervention manual and device operating range, to verify absence of an effective electromagnetic-field output while preserving matched sensory and procedural cues. The structured home exercise program described above will be identical in both groups and documented using participant exercise diaries; no resisted or progressive strengthening program will be implemented during the 4-week intervention period.
- Intervention Group (Active PEMF + Standardized Conservative Care): In addition to the standardized conservative care described above, participants in this arm will receive active electromagnetic-field delivery using the YS2002 magnetic thermo-vibration therapy device. The device will be operated at protocol-specified settings of 30 mT magnetic field intensity and a 40 Hz device frequency setting for 20 min once daily for 4 weeks. These active output settings are documented in the study intervention manual and will be confirmed during pretrial bench testing. As noted above, the settings were selected for biological plausibility, device reproducibility, and clinical feasibility rather than as an established optimal dose. The target is 28 sessions, and the prespecified minimum adherence threshold for per-protocol analysis is 20 completed sessions. The applicator will be positioned over the supraspinatus region with direct contact to the treatment area; a thin cloth layer may be interposed for comfort if needed. Thermal and vibration settings will be standardized and kept identical to those used in the sham group, so the trial contrast is active electromagnetic-field output under otherwise identical YS2002 sensory conditions. Staff will monitor skin reactions at each session and pause treatment if significant irritation occurs. Neither group will receive corticosteroid injections or other invasive treatments during the 4-week intervention period, and participants will be instructed to avoid seeking additional outside therapies for the injured shoulder until the Week 4 primary endpoint assessment is completed.

Throughout the 4-week treatment period, adherence to the interventions will be encouraged and recorded. Rehabilitation staff will record attendance for electrical stimulation and PEMF/sham sessions. NSAID usage will be tracked via patient diaries or pill counts. Participants will be educated about potential side effects of NSAIDs and device-based treatment (e.g., transient warmth, local skin irritation, or discomfort from prolonged positioning). If a participant in either group has significant pain exacerbation during the study, the treating physician may allow rescue analgesic medication (e.g., acetaminophen), and the reason, dose, and duration will be documented together with any other protocol-deviating co-interventions. Use of rescue analgesics will be summarized descriptively and may be considered in exploratory sensitivity analyses if imbalances between groups are observed.

### Outcome measures

All outcome measures will be assessed at baseline (Week 0, pre-treatment), end of treatment (Week 4, post-treatment), and follow-up at 3 months (Week 12), 6 months (Week 24), and 12 months (Week 52) after randomization, unless otherwise specified. The primary efficacy assessment will occur at Week 4. Week 4 was selected to capture early pain-related and functional response at the end of the intervention period; structural tendon healing will not be inferred from this endpoint.

Primary Outcome:—Constant-Murley Shoulder Score (CMS): The Constant-Murley score is a comprehensive clinical measure of shoulder function, ranging from 0 to 100 points (higher scores indicate better function) (26). It includes subjective pain (15 points) and activities of daily living (20 points) components, as well as objective range-of-motion (40 points) and strength (25 points) components. The score will be assessed using a standardized procedure informed by the original CMS method and the validated Chinese version (26, 27). Pain and activities are recorded based on patient report. Active range of motion will be measured with a goniometer, rotational components will be scored using predefined Constant test positions, and strength will be assessed using a calibrated force-measurement device in a predefined test position with the best of three trials recorded. The primary estimand for the trial is the adjusted between-group difference in Week 4 CMS. The Week 4 CMS effect will be interpreted as a short-term clinical effect, because symptomatic and functional improvement can occur before detectable biological tendon repair. The CMS will also be collected at 3, 6, and 12 months to describe recovery trajectory and durability as secondary analyses.

Secondary Outcomes:—Shoulder Pain Intensity: assessed using a 10-cm Visual Analog Scale (VAS; 0 = no pain, 10 = worst imaginable pain) for activity-related affected-shoulder pain during shoulder elevation or other usual provoking shoulder activity over the preceding 7 days at baseline, Week 4, and at 3, 6, and 12 months.—Shoulder range of motion (ROM): active shoulder flexion, abduction, and external rotation will be measured using the standardized goniometric protocol described below.—Shoulder Pain and Disability Index (SPADI): participants will complete the SPADI (28) using the validated Chinese version (C-SPADI) (29), scored according to standard instructions, at baseline, Week 4, and at 3, 6, and 12 months.—MRI structural outcomes at 6 months: tear location, maximal tear depth, maximal anterior-posterior tear length, estimated tendon-thickness involvement, and progression to full-thickness tear. If a pre-enrollment clinically indicated MRI is used as the baseline scan, it must include prespecified protocol-compatible sequences and minimum image quality criteria defined in the trial imaging manual; otherwise, a new baseline MRI will be obtained before randomization. Baseline tear-sidedness and tendon-thickness involvement will be recorded as prespecified structural descriptors and may be explored as prognostic covariates in sensitivity analyses. Because structural tendon remodeling is unlikely to be captured by the 4-week clinical endpoint and because the trial is not powered primarily for structural endpoints, MRI outcomes will be interpreted as exploratory.

ROM assessment protocol: Active ROM will be measured with a universal long-arm goniometer by a trained blinded assessor using standardized patient positioning and anatomical landmarks, consistent with published goniometric reliability literature (30, 31). Before each movement, participants will complete one practice trial. Three active pain-limited trials will then be recorded for each movement, with 30–60 s of rest between trials, and the mean of the three trials will be used for analysis. Flexion and abduction will be measured with the participant seated upright, feet flat, trunk stabilized, and compensatory trunk lean or shoulder hiking minimized. Flexion will be measured in the sagittal plane; abduction will be measured in the frontal/scapular plane according to the intervention manual; and external rotation will be measured with the shoulder adducted, the elbow flexed to 90 degrees, the forearm in neutral, and a towel roll between the elbow and trunk where needed. The same assessor, whenever feasible, will measure the same participant across visits, and the goniometer model, tested side, pain during measurement, and any protocol deviations will be recorded.

Safety and Other Measures:—Adverse Events: Non-serious adverse events occurring during the 4-week intervention period will be recorded systematically, whether related to treatment or not. We will specifically monitor potential adverse events attributable to PEMF/sham procedures (e.g., skin irritation or discomfort during therapy) and to NSAIDs (e.g., gastric upset). Serious adverse events and shoulder-related clinically relevant events during follow-up (up to 12 months) will also be documented, including hospitalizations or surgeries related to the shoulder. Adverse events will be categorized by severity and relatedness to the study intervention.—Drop-out Rate and Adherence: We will document discontinuation of study treatment, withdrawals from data collection, and losses to follow-up at each assessment point, along with reasons when available. Treatment adherence (attendance for electrical stimulation and PEMF/sham sessions, and medication compliance) will be tracked during the 4-week period.—Demographic and Baseline Data: Patient characteristics such as age, sex, duration of symptoms, baseline MRI structural measures, and relevant comorbidities will be recorded to describe the sample and, where appropriate, to support adjusted or sensitivity analyses.

Clinical outcome assessments (CMS, VAS, ROM, SPADI) will be performed by trained assessors at Week 4, 3 months, 6 months, and 12 months, ideally on the same day as a scheduled clinic visit (with the Week 4 assessment scheduled at 28 ± 3 days after randomization and predefined visit windows for later follow-up). Assessors will be blinded to group allocation as described above. MRI scans will be anonymized and coded before reading. Baseline and 6-month MRI scans will be reviewed independently by two trained musculoskeletal readers who are blinded to treatment allocation and clinical outcomes, using a predefined imaging manual and calibration cases before formal reading. The manual will define tear location (articular-sided, bursal-sided, or intrasubstance), maximal tear depth, maximal anterior-posterior tear length, local tendon thickness, estimated percentage tendon-thickness involvement, and progression to full-thickness tear. Maximal tear depth and tendon thickness will be measured on the slice showing the greatest partial-thickness defect, tear length will be measured along the anterior-posterior extent of the involved supraspinatus tendon, and percentage tendon involvement will be calculated as tear depth divided by local tendon thickness. Continuous measurements will be recorded to the nearest millimeter where feasible and percentage involvement to the nearest 5 percentage points. The mean of the two reader measurements will be used when readings are within prespecified tolerance limits; discrepancies exceeding 2 mm for tear depth or length, 10 percentage points for percentage involvement, or any disagreement on full-thickness progression will be resolved by consensus or adjudication by a third senior reader. Inter-rater agreement will be summarized using intraclass correlation coefficients for continuous structural measures and kappa or weighted kappa statistics for categorical MRI variables.

### Statistical methods

A detailed statistical analysis plan will be finalized prior to database lock and before unblinding of group codes. Efficacy analyses will be conducted on an intention-to-treat (ITT) basis, including all randomized participants analyzed according to their allocated group regardless of adherence. A per-protocol (PP) analysis may be performed as a sensitivity analysis and will include participants who complete at least 20 of the 28 planned YS2002 sessions, do not receive prohibited co-interventions before the Week 4 primary endpoint assessment, and have no major post-randomization eligibility violation or treatment-allocation error.

Categorical variables will be presented as frequencies and percentages. Baseline characteristics will be summarized descriptively by randomized group. No hypothesis testing of baseline differences will be performed.

Week 4 participant guesses about treatment allocation will be summarized by randomized group as counts and percentages. Where cell counts permit, Bang blinding index values with 95% confidence intervals will be calculated separately for the active and sham groups. Values close to zero will be interpreted as consistent with successful blinding, whereas positive values will suggest correct identification above chance. This analysis will be considered a methodological assessment of masking rather than an efficacy endpoint (25).

For the primary efficacy endpoint, the primary estimand will be the adjusted between-group difference in Week 4 Constant-Murley score in the intention-to-treat population. This comparison will be performed using analysis of covariance (ANCOVA), with Week 4 CMS as the dependent variable, treatment group as the main factor, and baseline CMS as a covariate. Effect sizes will be reported as adjusted mean differences with 95% confidence intervals.

For secondary outcomes collected repeatedly over time (VAS, ROM, SPADI, and CMS trajectory), we will use linear mixed-effects models with fixed effects for group, time, and group-by-time interaction to evaluate between-group differences in trajectories from baseline through 12 months. Continuous MRI structural outcomes at 6 months, including maximal tear depth, maximal anterior-posterior tear length, and estimated tendon-thickness involvement, will be analyzed using ANCOVA adjusting for the corresponding baseline MRI measure. The primary MRI analysis will use the mean of the two independent reader measurements when agreement is within tolerance limits and the consensus or adjudicated value when discrepancies exceed prespecified thresholds. Binary or categorical MRI outcomes, including progression to full-thickness tear and tear-location category, will be summarized descriptively and compared between groups using chi-square tests or Fisher's exact tests as appropriate. Inter-rater reliability will be summarized for key MRI variables before consensus/adjudication. Because of limited power and measurement variability for structural endpoints, all MRI analyses will be interpreted as exploratory. Categorical outcomes (e.g., occurrence of adverse events; receipt of corticosteroid injection or surgery during follow-up) will be compared using chi-square tests or Fisher's exact tests as appropriate. Time-to-event analyses for additional intervention or surgery will be exploratory and reported only if event numbers are sufficient.

All hypothesis tests will be two-sided, and a *p*-value < 0.05 will be considered statistically significant. We do not plan to adjust for multiple comparisons for secondary outcomes, as these analyses are exploratory; the primary outcome will be the main determinant of efficacy. No confirmatory subgroup analyses are planned because of the modest sample size; any subgroup analyses undertaken will be clearly labeled exploratory. Data analysis will be performed using IBM SPSS Statistics version 26.0 (IBM Corp., Armonk, NY, USA).

We will check model assumptions (normality, homogeneity of variance) for parametric analyses. For repeated-measures analyses, mixed models inherently accommodate missing data under a missing-at-random assumption. If Week 4 CMS is missing, the primary ANCOVA analysis will use multiple imputation under a missing-at-random assumption; the imputation model will include treatment group, baseline CMS, age, sex, symptom duration, and prespecified predictors of missingness and outcome. Complete-case analysis will be performed as a sensitivity analysis. Interim analysis is not applicable given the modest sample size; all analyses will occur after completion of follow-up and database lock.

### Data management

Data will be recorded on paper case report forms and entered into a password-protected electronic database by trained study staff. Range checks and logic checks will be applied to improve data quality, and key variables will be cross-checked against source documents. The database will be stored on a secure hospital server with regular backups. Only authorized members of the research team will have access to identifiable information; all analyses will be performed on de-identified datasets.

### Data monitoring and safety oversight

Given the noninvasive nature of PEMF and the short 4-week intervention period, a formal independent data monitoring committee is not planned. The principal investigator will oversee trial conduct and safety. Non-serious adverse events will be collected systematically during the 4-week intervention period. Serious adverse events and shoulder-related additional interventions will be collected throughout the 12-month follow-up and reported to the ethics committee in accordance with institutional requirements. An internal monitoring process will be conducted at prespecified intervals by designated members of the trial management group. This monitoring process is not independent of the investigators or the sponsor. Consent forms, adherence logs, and completeness/consistency of outcome data will be reviewed, and any protocol deviations will be documented and addressed through retraining or corrective actions.

### Protocol access and data sharing

Protocol access: This protocol will be publicly available via this publication and the trial registry record (32). The detailed statistical analysis plan will be finalized before database lock and prior to unblinding, and will be made available on reasonable request.

Data sharing: After completion of the trial, the final de-identified trial dataset, data dictionary, and statistical code will be accessible to the principal investigators and authorized members of the study team responsible for data management and analysis. After publication of the primary trial report, de-identified individual participant data, the data dictionary, the statistical code, and the statistical analysis plan will be available from the corresponding author on reasonable request for methodologically sound proposals, subject to ethics approval, institutional approval, and a data-sharing agreement. Data will be available for 5 years after publication of the primary report. There are no contractual agreements limiting investigators' access to the final trial dataset.

Dissemination: Results will be disseminated through peer-reviewed publications, academic conference presentations, and updates to the trial registry. A brief summary of the results will be provided to participants upon request.

## Discussion

This trial is designed to evaluate the incremental effect of active electromagnetic-field delivery within the YS2002 magnetic thermo-vibration therapy device when added to standardized conservative care in adults with symptomatic MRI-confirmed partial-thickness supraspinatus tears. All participants receive a multimodal conservative-care package; therefore, a significant result would support an additional contribution of active electromagnetic-field output over matched sham output within this standardized package, not the independent effect of standalone PEMF without concomitant rehabilitation. By focusing on a defined MRI-confirmed population and by standardizing co-interventions across groups, the study aims to clarify the specific contribution of active electromagnetic-field delivery within the YS2002 treatment platform.

Several limitations should be acknowledged. First, this is a single-center trial with a modest sample size (*n* = 50). It is powered for a relatively large 10-point Week 4 CMS difference and may be insufficient to detect modest clinical effects; longer-term outcomes and MRI structural endpoints are especially underpowered, so null structural findings will not exclude smaller treatment effects. Second, optimal PEMF dosing for rotator cuff pathology is not established. Although the selected 30 mT, 40 Hz, 20-minute daily regimen is justified by device reproducibility, clinical feasibility, and related preclinical/clinical shoulder evidence, it should not be interpreted as an optimized or universally generalizable dose. Third, the intervention is embedded in a standardized multimodal conservative-care package that includes medication when indicated, electrical stimulation, activity modification, home exercise, and matched YS2002 heat and vibration. Consequently, this trial estimates the incremental effect of active electromagnetic-field delivery over sham electromagnetic-field delivery within that package; it cannot determine the independent efficacy of standalone PEMF compared with no rehabilitation or with each individual co-intervention removed. Fourth, the Week 4 primary endpoint is intentionally an early clinical endpoint. Tendon remodeling and structural healing are unlikely to be fully captured within 4 weeks, and any favorable Week 4 finding will be interpreted mainly as symptomatic and functional improvement rather than biological tendon repair. Fifth, MRI structural measurements such as tear depth, anterior-posterior length, and percentage tendon involvement may be subject to interobserver variability, particularly for partial-thickness tears (33). We have therefore prespecified an imaging manual, independent blinded duplicate readings, discrepancy thresholds, consensus/adjudication procedures, and reliability statistics; nevertheless, the 6-month MRI analyses remain exploratory. Sixth, the relatively homogeneous population defined by the eligibility criteria, including the age range, symptom-duration limit, baseline activity-related pain threshold, supraspinatus-only involvement, and tear-length restriction, enhances internal validity but limits generalizability to patients outside these criteria, including younger or older patients, chronic tears, minimally painful tears, tears larger than 3 cm, or multi-tendon involvement. Finally, complete blinding of treatment providers is not feasible despite participant sham control and blinded outcome/MRI assessment.
